# Scientific approaches to defining HPV vaccine‐induced protective immunity

**DOI:** 10.1002/ijc.35345

**Published:** 2025-02-13

**Authors:** Matti Lehtinen, Pierre van Damme, Simon Beddows, Ligia A. Pinto, Filipe Mariz, Penelope Gray, Joakim Dillner

**Affiliations:** ^1^ Department of Vaccines Institute for Health and Welfare Helsinki Finland; ^2^ Center for Cervical Cancer Elimination, Department of Clinical Science, Intervention and Technology Karolinska Institute Stockholm Sweden; ^3^ Centre for the Evaluation of Vaccination@Vaccinopolis Universiteit Antwerp Antwerp Belgium; ^4^ Virus Reference Department, Public Health Microbiology Division UK Health Security Agency London UK; ^5^ HPV Serology Laboratory Frederick National Laboratory for Cancer Research Frederick Maryland USA; ^6^ Division of Infections and Cancer Deutsches Krebsforschungszentrum Heidelberg Germany

**Keywords:** antibody, cancer, hepatitis B virus, human papillomavirus, vaccine

## Abstract

Seventeen years after the licensure of prophylactic human papillomavirus (HPV) L1 virus‐like‐particle vaccines, a defined antibody level that correlates with vaccine‐induced protection against HPV infections and associated neoplasia is missing. In contrast, correlates of protection have been defined for many viral vaccines, including for the hepatitis B virus (HBV) vaccine. This review includes lessons learned from vaccination against HBV and the use of an established protective HBV surface antigen antibody level: 10 mIU/mL, an overview of HPV infection‐induced and HPV vaccine‐induced antibody responses, successful efforts to establish international standardization of serological reagents and associated tools, and 15‐year vigilance of HPV vaccine‐induced antibody levels in a vaccination cohort against breakthrough infections. This report identifies progress but also gaps on the journey toward the definition of a HPV vaccine‐induced correlate of protection.

## Graphical abstract

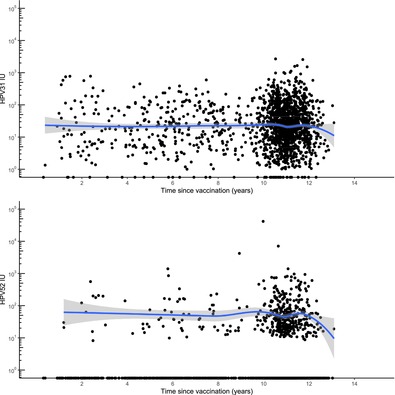

## 1 INTRODUCTION

Successful phase III trials resulted in the licensure of prophylactic human papillomavirus (HPV) vaccines in 2006/7,^1^, ^2^ but the definition of protective HPV antibody level(s) is pending. Vaccine‐induced correlate of protection against chronic infection with hepatitis B virus (HBV): an HBs antibody level ≥10 mIU/mL, was established in 1999.^3^

There are lessons from HBV vaccinology with regard to (1) vaccine doses needed or the multiplicity of HBV surface antigens (HBsAg) required to establish the protective antibody level,^4^ (2) the combination of a virus‐like‐particle (VLP) vaccine with other licensed vaccines,^5^, ^6^ and (3) sustainability of protection without detectable protective vaccine‐induced antibody levels (Table 1).^7^, ^8^ Protective T‐helper cell‐augmented and B‐memory cell‐derived anamnestic antibody response is effective against HBV challenge decades after full‐scale immunization, even in the absence of boosters after the primary series.^9^ However, it is unclear to what extent these lessons are applicable for HPV vaccine‐induced protection.

**TABLE 1:** Comparison of hepatitis B virus (HBV) and human papillomavirus (HPV) vaccines and vaccination.

Parameter	HBV vaccine	HPV vaccine
Infection	Systemic, liver	Mucosal
Prevented malignancies	HCC	Anogenital and oropharyngeal carcinoma
Immunogen	HBV‐sAg	Major capsid protein L1
Valency	1 (3^a^)	2^b^, 4^c^, 9^d^
Regimen	3+ doses	1–3 doses
Correlate of protection established	Yes	No
Antibody correlate	10 IU/mL	N/A
Adjuvant	Alum	Alum and AS04^e^
High antibody levels	Yes	Yes
Durability (years)	30+	15+
Vaccine response more robust than natural infection	Yes	Yes
International standardization of laboratory markers	Yes	Yes
Vaccine‐type breakthrough infections	Yes	Little evidence
Protection established in animal challenge experiments	Yes	Yes

Abbreviation: HCC, hepatocellular carcinoma.

^a^ HBsAg, preS1, and preS2.

^b^ Bivalent (HPV16/18).

^c^ Quadrivalent (HPV6/11/16/18).

^d^ Nonavalent (HPV6/11/16/18/31/33/45/52/58).

^e^ Bivalent vaccine.

VLP‐based HPV vaccines mimic viral capsids including the repetitive surface‐exposed antigenic determinants and make excellent immunogens.^9^, ^10^ The immune response against VLPs includes high levels of neutralizing antibodies that protect against HPV infection and subsequent disease. Vaccine‐induced antibody levels are often used as a noninferiority proxy for efficacy, but an antibody correlate of protection is missing for HPV vaccination. Investigations are ongoing on the specificity of the antibodies generated including: resolution of the antibody repertoires created following vaccination and natural infection, appreciation of the durability of long‐lived plasma cells and associated antibody products including those with cross‐reactivity, and vigilance for changes to lineage‐specific ecological distribution following vaccine deployment.

International standardization of reagents and associated tools for HPV serology is a prerequisite for the definition of vaccine‐induced protective HPV antibody levels. This activity was established in 2006 following a World Health Organization (WHO) initiative^11^, ^12^ leading to the creation of International Standards (IS). With the introduction of one‐dose vaccination regimen, this is even more important^13^, ^14^ and forms a basis for the quest to determine HPV vaccine‐induced protective antibody levels.

The HPV type‐ and lineage‐specific differences in natural infection‐derived neutralizing capsid antibodies may help to put the HPV VLP vaccine‐specific differences in neutralizing antibody responses into perspective. Low or absent quadrivalent and nonavalent vaccine‐induced HPV18 antibody levels have been confirmed by independent laboratories and methods.^15^, ^16^, ^17^, ^18^ The sequences of HPV16 and 18 L1 genes used in the bi‐ and quadrivalent vaccines are essentially similar (bivalent vaccine VLPs have a short N‐terminal truncation), but difference in their expression systems (yeast/eukaryotic) might result in different folding, posttranslational modification, and availability of antigenic B‐cell epitopes.

Excellent vaccine efficacy (VE) and the lack of type‐restricted breakthrough infections and/or lesional end points have hampered the identification of vaccine‐induced protective HPV antibody levels. Significant correlation between bivalent HPV16/18 vaccine‐induced cross‐neutralizing antibody levels and VE against infections with nonvaccine HPV types provides possibilities to determine protective antibody level(s).^19^

## 2 HEPATITIS B VACCINE‐INDUCED PROTECTIVE IMMUNITY

Hepatitis B is caused by the HBV. HBV is primarily infecting hepatocytes, and cellular immune response to viral proteins in infected hepatocytes causes liver damage. Infection with HBV causes a broad disease spectrum, including subclinical infection, acute, clinically overt self‐limited hepatitis, and fulminant hepatitis. The average incubation period is 90 days from exposure to onset of jaundice and 60 days to onset of abnormal liver tests.

Persons infected with HBV can develop a chronic infection, which can lead to liver cirrhosis or hepatocellular carcinoma (HCC).^20^, ^21^, ^22^ The risk of developing a chronic infection varies inversely with age of HBV acquisition: Up to 90% of infants infected during the 1st year of life develop chronic infection, compared with 30% of children infected between the ages 1 and 4 years and less than 5% of persons infected as adults. Persons who have persistence of HBsAg in serum for at least 6 months are classified as having chronic infection and defined as carrier of HBV.^23^

HBV can spread through contact with infected body fluids like blood, saliva, vaginal fluids, and semen. It can also be passed perinatally.^25^ In low‐endemicity areas, most infections occur in relatively well‐defined risk groups: blood product and solid organ transplantation recipients, dialysis patients, prisoners, iv drug‐users, household and sexual contacts of people with chronic HBV infection, people with multiple sexual partners, and healthcare providers. In high‐endemicity areas, a high proportion of infections occur as a consequence of perinatal and early childhood exposures, which can more likely lead to chronic infection.^25^

HBsAg prevalence ranges from extremely low, less than 0.1% in western/northern/central Europe to 6%–8% in some countries of eastern Europe and Central Asia. WHO estimates that 254 million people were living with chronic hepatitis B infection in 2022, with 1.2 million new infections each year. In the WHO European Region, there are 13.3 million HBV carriers, leading to approximately 36,000 deaths per year from HBV‐related HCC and cirrhosis.^26^ In 2022, hepatitis B resulted in an estimated 1.1 million deaths, mostly from cirrhosis and HCC. The lifetime risk for HCC in a chronically infected person is 10%–25%, which is 15–20 times greater than that for persons without HBV infection.^24^, ^25^

First vaccines were based on HBsAg (the 22‐nm particle) derived from plasma of persons with chronic HBV infection. However, these have been replaced by recombinant by recombinant HBsAg vaccines, that offer unlimited supply. They contain 2.5–40 μg of HBsAg protein and most often a generic adjuvant: aluminum phosphate or aluminum hydroxide.^25^ In contrast to the wild‐type HBV envelope proteins comprising the first vaccines, yeast‐derived S‐particles in currently used vaccines are not glycosylated while the more immunogenic recently introduced mammalian cell‐derived envelope protein vaccines are.^27^

Plotkin defines a correlate of protection as “an immune function that correlates with and may be biologically responsible for vaccine‐induced‐efficacy…although from a biological point of view vaccines produce a variety of protective functions, some are more important than others and are useful to predict efficacy.”^28^ An anti‐HBs concentration of 10 mIU/mL or more measured 1–3 months after administration of the last dose of a complete vaccination series is considered a reliable marker of protection against infection and disease; 10 mIU/mL is the “minimum protective level” and defined as the lowest concentration of vaccine‐induced antibodies to serve as the biomarker for induction of strong memory B‐cell response.^9^, ^29^, ^30^, ^31^

The level of 10 mIU/mL is an arbitrary level, established in a conventional way, as a result of a critical analysis of data regarding natural infection, passive immunity, and vaccine trials.^3^, ^9^, ^29^, ^30^, ^31^, ^32^ Virtually complete protection (for decades) against both acute and chronic infection was observed in immunocompetent persons who developed anti‐HBs concentrations of >10 mIU/mL after vaccination, even if over time anti‐HBs concentrations declined to less than 10 mIU/mL. The protective efficacy of hepatitis B vaccination is based on the induction of anti‐HB antibodies and on that of memory B and T cells.^33^ Routine postvaccination testing for immunity is recommended only for high‐risk persons whose clinical management depends on knowledge of their immune status.^29^

Several hepatitis B vaccine schedules exist but are usually composed of two priming doses and a third dose with an interval of 6 months after the first one: These schedules induce levels of seroprotection (≥10 mIU/mL) of 95% and more in newborns, infants, schoolchildren, and adolescents. This percentage will be close to 90% in healthy adults. Over time, vaccine‐induced anti‐HBs concentrations decline rapidly within the first year and more slowly thereafter. However, protection has been shown to outlast the presence of vaccine‐induced antibodies, offering long‐term protection against acute disease and development of HBsAg carriage for a minimum of at least 30 years.^27^, ^30^, ^34^, ^35^ Stimulated memory cells have ample time to produce effective levels of anti‐HBs after exposure to and invasion by HBV, since this process takes only a few days^36^ and the incubation period of hepatitis B is long.^34^, ^36^, ^37^

The WHO, as well as advisory groups in the United States and Europe, do not recommend routine booster doses of HBV or HPV vaccines, or periodic serological testing to monitor anti‐HBs concentrations for immunocompetent persons who have responded to vaccination or in universal immunization programs, based on currently available scientific evidence.^25^, ^30^, ^38^

The focus of the hepatitis B vaccination programs was in the 1980s on groups at risk for hepatitis B, for example, healthcare providers and men who have sex with men. Impact can be expected at individual level but not at public health level. In the 1990s, universal immunization programs against hepatitis B started, targeting newborns, infants, and young children; 189 countries had integrated hepatitis B vaccine into their national childhood immunization systems by the end of 2019. Worldwide coverage of hepatitis B third vaccine dose is estimated at 80%–85%.^40^ The impact of these large immunization programs is demonstrated by the decrease in the burden of hepatitis B and HBV‐related diseases.^25^, ^39^, ^40^

The World Health Assembly adopted in May 2016 the first “Global health sector strategy on viral hepatitis, 2016–2020.” The strategy sets targets that align with those of the Sustainable Development Goals and highlights the critical role of universal health coverage. The aim is to eliminate viral hepatitis as a public health problem; the global targets are the reduction of new viral hepatitis infections by 90% and deaths due to viral hepatitis by 65% by 2030.^41^, ^42^, ^43^, ^44^ In order to do so, vaccination programs need to be incorporated in existing mother and child care programs, and integrated in an overall plan including vaccination and therapy. For this to work, collaboration between all stakeholders needs to be enhanced, within and with ministries of health, partners of civil societies, and patient groups.

## 3 MAGNITUDE AND SPECIFICITY OF HPV INFECTION AND VACCINE‐INDUCED NEUTRALIZING ANTIBODIES

Prophylactic HPV vaccines comprise VLPs that mimic the viral capsids of a range of types and demonstrate remarkable efficacy against type‐specific infection and disease. The detection of vaccine‐type neutralizing antibodies in the serum and genital secretions of vaccinees, together with the passive transfer of protections in animal models, has indicated that vaccine‐induced protection is primarily mediated by neutralizing antibodies.^9^ Neutralizing antibodies are thought to prevent binding of the capsid to heparan sulfate proteoglycans located within the basement membrane rather than at the cell surface or interfere with a postattachment event.^45^ Seroconversion following vaccination nears 100% with the resulting antibody levels seemingly orders of magnitude in excess of the level required for protection. Vaccination induces a robust B‐cell memory that can be recalled many years later following vaccination with a booster dose, but the protective antibodies that persist for at least a decade, remarkably stable even following a single dose of vaccine, are likely due to the induction of long‐lived plasma cells.^9^, ^46^ In contrast, HPV infection occurs at a mucosal site without inflammation or viremic stage thereby providing little exposure to host immune surveillance and allowing infection to persist for many months before being cleared by cell‐mediated immunity. Seroconversion is only observed for a proportion of natural infections, and the resulting serum antibody titers are low, though may reduce the risk of subsequent type‐specific infections.^47^ Vaccine‐induced antibodies are of a high magnitude and exhibit specificities that also provide low‐level cross‐protection against related nonvaccine incorporated types, a phenomenon not seen with natural infection‐induced antibodies.^9^

HPV serology uses intact capsids in either a ligand‐binding assay (enzyme‐linked immunosorbent assay [ELISA]), a monoclonal antibody (MAb) competition assay (cLIA), or along with a reporter protein, pseudovirus‐based neutralization assay.^48^ The ELISA measures the total capsid‐specific antibody binding fraction within a serum sample, while the cLIA highlights a subset of antibodies whose specificities overlap with a representative neutralizing MAb, and the pseudovirus‐based neutralization assay quantifies the antibody fraction capable of preventing (pseudo)virus entry into susceptible cells in vitro. The ELISA can also be used to estimate antibody avidity, which may distinguish between natural infection and vaccine‐elicited binding antibodies, while the cLIA highlights overlap between the specificity of natural infection and vaccination‐derived antibodies. Enumeration of type‐specific peripheral blood memory B cells found a refocusing of the antibody repertoire from a “binding antibody phenotype” during natural infection to a “high‐affinity neutralizing antibody phenotype” following one dose of vaccine.^46^

The HPV VLP is an icosahedral lattice comprising 72 pentamers of the major capsid protein (L1), and the repetitive arrangement of the L1 pentamers is a key contributor to its remarkable immunogenic properties. Mutagenesis and cryoelectron microscopy have mapped the epitopes of several murine MAbs to the L1 capsid surface including their dependency on residues within the surface‐exposed external loops (BC, DE, EF, FG, and HI).^49^ Neutralizing antibody response elicited following natural infection or vaccination is also directed against these surface‐exposed loops although the specific antibody profiles may differ.^50^ The neutralizing antibodies are almost exclusively type‐specific in their reactivity due to loop‐specific amino acid residue variation between different types.^51^ However, limited cross‐reactivity can be seen between genetically related types where the external loop residues share some degree of sequence or structural homology (e.g., between HPV16/31 and HPV18/45) which likely explains the limited cross‐protection seen against these nonvaccine types (HPV31/45) in vaccine trials and real‐world settings.^9^, ^46^ The reduced seroconversion rates and protection against nonvaccine HPV types may provide a context in which to establish a correlate of HPV vaccine‐induced protection.

HPV exists as naturally occurring lineage and sublineage variants, although the consequences of this diversity for viral structure and function are unclear.^52^ Pseudoviruses representing this lineage diversity exhibited differential recognition by antibodies (including antibodies against nonvaccine types) elicited following natural infection and derived by vaccination. The antigenically distant lineages tended to be ones with the greatest sequence distance from lineage A (the reference sequence).^53^, ^54^ This differential recognition was attributed to residues in the external surface‐exposed loops and was of a sufficient magnitude to suggest that some lineages should be considered antigenically distinct within their respective types. These data suggest a degree of overlap in natural infection and vaccine‐induced antibody repertoires, and highlight the importance of the loops in determining both type‐specific and lineage‐specific capsid antigenicity. Trial data suggest differential vaccine efficacies against some nonvaccine HPV type lineage variants^55^ and may provide additional context for defining antibody correlate of protection against the nonvaccine types. Surveillance of lineage distribution in some settings may assist vigilance against potential lineage‐specific ecological replacement following vaccine deployment. Understanding the magnitude, breadth, specificity, and durability of immune response elicited by HPV vaccines compared to natural immunity improves the evidence base of this public health intervention.

## 4 INTERNATIONAL STANDARDIZATION ON HOW TO EVALUATE HPV VACCINE‐INDUCED RESPONSES

HPV serology is essential for evaluation of vaccine immunogenicity and natural infection. As HPV prophylactic vaccine trials are increasingly using serologic end points for licensure of new indications or products, standardization to ensure that generated data can be reliably compared across different studies to inform public health decisions is important. WHO‐established standards and controls are critical for data harmonization. Following a 2001 international workshop organized by Harald zur Hausen, WHO launched a program toward international standardization of HPV serology, including establishing IS against the major oncogenic HPV types.^11^, ^12^

In 2017, the efforts were continued by the HPV Serology Laboratory (HPVSL), led by Dr. Ligia Pinto with support from National Cancer Institute and the Bill & Melinda Gates Foundation.^56^, ^57^ Recent progress includes (1) additional development of IS and of secondary standards, (2) proficiency panels for the nine HPV types targeted by currently approved vaccines, (3) production of critical reference serology reagents (e.g., HPV VLPs), (4) development and validation of multiplex high‐throughput assays, and (5) assay guidelines and standard operating procedures.

Standards, proficiency panels, and serological grade VLPs for the nine HPV types targeted by currently licensed vaccines were produced and made available to the community either through the FNLCR or NIBSC websites: (https://frederick.cancer.gov/research/vaccine-immunity-and-cancer-directorate/hpv-serology-laboratory) and (https://nibsc.org/products/brm_product_catalogue/sub_category_listing.aspx?search=antihuman%20papillomavirus).^58^, ^59^ In addition, the HPVSL developed and validated a 9‐plex Luminex‐based serology assay and partnered with the WHO to publish a Serology Secondary Standards manual,^60^, ^61^ and aims to develop and validate highly sensitive/specific assays to detect low levels of vaccine‐induced antibodies against HPV types of interest.

**FIGURE 1:**
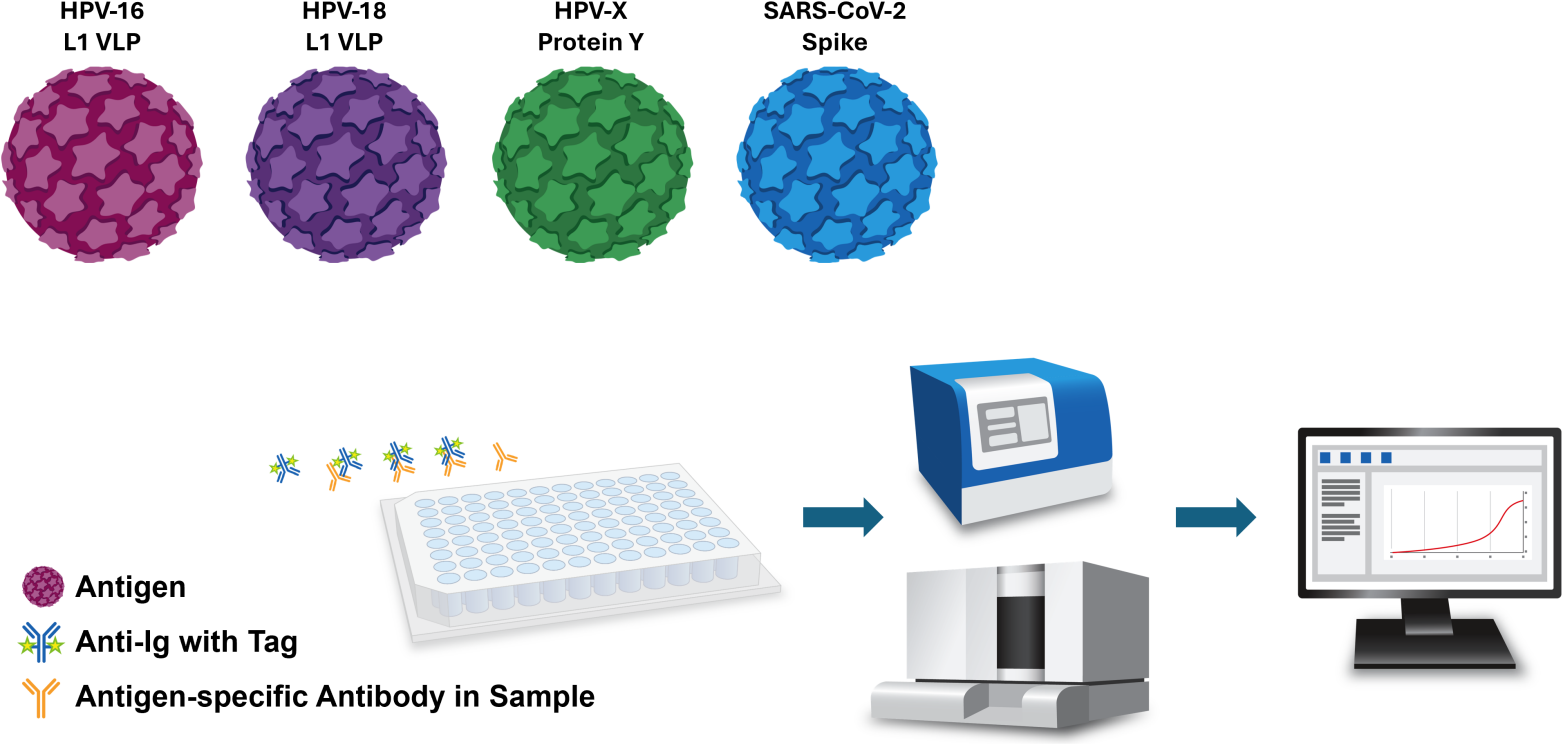
Plug‐and‐play enzyme‐linked immunosorbent assay. Plug‐and‐play serology technology allows for accurate, reliable, and customizable antibody level measurement for different human papillomavirus (HPV) types, proteins, or even different viruses such as SARS‐CoV‐2.

HPVSL has developed a plug‐and‐play ELISA that enables accurate and customizable serology analysis, targeting different antigens (Figure 1). This technology has the potential to push serology applications and standardization to include any HPV (or any other virus) type as new vaccines seek to target additional HPV types. Specifically, a target antigen of interest can be coated onto the ELISA plate (or conjugated to beads in the case of the Luminex‐based assays), allowing for measurement of the level of binding antibodies in patient serum, plasma, or other biospecimen. This can be done in a single‐plex format to screen multiple samples for antibodies against the target antigen or in a multiplex format using bead technology to screen for antibodies against multiple antigens in a single sample.

The serological assays developed at HPVSL have been implemented in a number of HPV vaccine trials including: ESCUDDO (NCT03180034), PRISMA ESCUDDO (NCT05237947), and PRIMAVERA (NCT03728881) in Costa Rica, as well as the prime and deferred booster dose schedule trial (NCT02568566) in the United States, DoRIS (Dose Reduction Immunobridging Study; NCT02834637) in Tanzania, KEN SHE (NCT03675256) in Kenya, and HOPE (HPV One‐ and Two‐Dose Population Effectiveness study) in South Africa.^62^, ^63^, ^64^

The international HPV Serology Standardization has contributed in part to the recent WHO endorsement of the one‐dose HPV vaccine schedule in 2022. The HPVSL is developing training materials and promoting assay transfer and standardization in HPV laboratories across the world, with more than 47 agreements established globally. Moving forward, HPVSL and collaborators are developing new standards and assays for additional HPV types that will be included in future vaccines, and a multiplex pseudovirion neutralization assay. The standardized tools developed can be applied to identify correlates of protection and minimal antibody levels of protection using longitudinal and breakthrough specimens.^65^

## 5 QUANTITATIVE AND QUALITATIVE DIFFERENCES IN VACCINE‐INDUCED L1 ANTIBODY RESPONSES

HPV immunization programs will soon provide VLP‐based high‐valency vaccines protecting against all the major cervical cancer‐causing genotypes in a global scale,^66^ and novel vaccines will be licensed on the basis of immunobridging studies comparing anti‐L1 antibody concentrations across different vaccine cohorts. It would also be important to define vaccine‐induced correlates of protection, an immune function that correlates with VE^28^ against both HPV infection and neoplasia.

Both bi‐ and quadrivalent vaccines induce, at peak immunogenic interval (1–2 months after immunization), seroconversion for HPV16 and HPV18 antibodies in almost all vaccinees of the trials that resulted in vaccines' licensure.^67^ The robust seroconversion rates observed were, however, accompanied by distinguishable features. Neutralizing, cross‐neutralizing, and VLP‐binding antibodies are found in higher concentrations and more prevalently in bivalent vaccine recipients as compared to quadrivalent vaccine recipients in different cohorts including the adolescent U.K. girls, Finnish girls participating the PATRICIA (bivalent) vaccine trial, and Indian girls participating the IARC (quadrivalent) vaccine trials.^68^, ^69^ These differences may reflect the quality of vaccine‐induced antibody responses. In Finnish vaccine recipients followed up for 2–12 years postvaccination, sustained HPV16/18 antibody seroprevalence rates were found in all bivalent vaccine recipients, but 10%–18%^70^ and 14%^17^ of quadrivalent vaccine recipients had no L1‐binding or neutralizing HPV18 antibodies. There is so far no evidence of inadequate protection against HPV18‐associated lesions in these women.

The DoRIS trial has reported essentially similar seroprevalence rates and avidity indexes for both HPV16/18 antibodies 2 years postvaccination in recipients of 1, 2, or 3 doses of either the bi‐ or the nonavalent vaccines.^71^, ^72^ Vaccine‐induced antibody avidity seems to be influenced by natural infection. Tsang and colleagues^73^ showed that HPV16 antibodies elicited in vaccinees of the Costa Rica Vaccine Trial displayed lower avidity up to 11 years postvaccination in baseline HPV16‐seropositives compared to HPV16‐seronegatives, regardless of the regimen (1 or 3 doses). The monitoring of vaccine‐induced avidity in the single‐ and multiple‐dose cohorts is warranted.

In spite the major role of neutralizing antibodies in preventing HPV infection, it is important to note that vaccine‐induced correlates of protection against infection and lesions are multiple and synergistic. Beyond neutralization, antibodies can also exert antiviral, Fc‐effector functions. Roy et al.^74^ demonstrated that both bi‐ and quadrivalent vaccines can induce strong and diverse Fc‐effector functions up to 1 year postvaccination in adult recipients of three‐dose regimen, which included antibody‐dependent complement deposition, and cellular and neutrophil phagocytosis. Notably, lower HPV18‐specific Fc‐effector functions were observed in quadrivalent than in bivalent vaccine recipients.

## 6 VACCINE‐INDUCED ANTIBODY LEVELS CONFERRING PROTECTION AGAINST HPV INFECTIONS/LESIONS

Licensed HPV vaccines have very high VEs,^75^, ^76^ and only few breakthrough cases have been documented with misclassification owing to viral depositions or an infection acquired prevaccination likely responsible.^77^ HPV vaccine trials on one‐ versus three‐dose regimen have reported equally high VEs against vaccine‐type HPV infections,^78^, ^79^ but against HSIL end point the three‐dose regimen was reported to be superior in a unique registry‐based long‐term follow‐up study.^80^ Thus, immune correlates of protection against various end points are lacking.

Among the bivalent and quadrivalent HPV vaccine recipients, two‐ and three‐dose vaccine‐induced HPV16/18 antibody levels are higher than among one‐dose recipients at the peak immunogenicity interval. Overall, the antibody levels reach a sustainable plateau over time, albeit at different levels.^18^, ^68^, ^71^, ^72^ At low antibody levels, protection from HPV infection was conferred in mice at levels 100‐fold below the available neutralization assay's limits of detection^9^, ^81^, ^82^, ^83^ possibly via vaccine‐induced antibody‐dependent phagocytosis (not a large‐scale analysis).

The bivalent and quadrivalent HPV vaccines have varying cross‐protective VEs against HPV types phylogenetically related to HPV16/18 and induce sustainable cross‐neutralizing antibody levels in different proportions.^18^, ^84^, ^85^ In bivalent vaccine recipients, the seroprevalence to clade A9 HPVs was correlated with VEs against HPV16/31/33/35/52/58.^18^ In an observational study after bivalent vaccination, HPV52 antibody level was inversely associated with the risk of subsequent HPV52 infection.^86^

A community‐randomized trial (NCT00534638) was initiated by the Finnish Institute for Health & Welfare in 2007.^87^, ^88^ In total, 12,402 females received bivalent HPV16/18 vaccination at the age of 12–15 years during the initial stages of the trial and a further 4564 females, who initially received HBV vaccination, received HPV16/18 vaccination at age 18 years. They continued in a screening study at the ages of 22, 25, and 28 years.^89^ Between 2019 and 2024, 6958 participants of this study donated two blood samples. Additional blood samples from the participants were collected from the Finnish Maternity Cohort.^90^ To be eligible for inclusion in the protective antibody levels' study, the participant must have given at least two cervicovaginal samples and have a serum sample prior or on the same date as the first (HPV DNA‐negative) screening sample. We evaluate whether the HPV16/18 vaccine‐induced cross‐reactive L1‐binding antibodies to clade A9 and A7 HPV types correlate with the risk of any of these HPV infections postvaccination. Sera are analyzed for the binding IgG to HPV6/11/16/18/31/33/35/39/45/51/52/56/58/59/68/73 using a multiplexed heparin‐bound HPV L1/L2 pseudovirion Luminex assay.^91^ Antibody levels are in international units (IU) for HPV 6/11/16/18/31/33/45/52/58.

**FIGURE 2:**
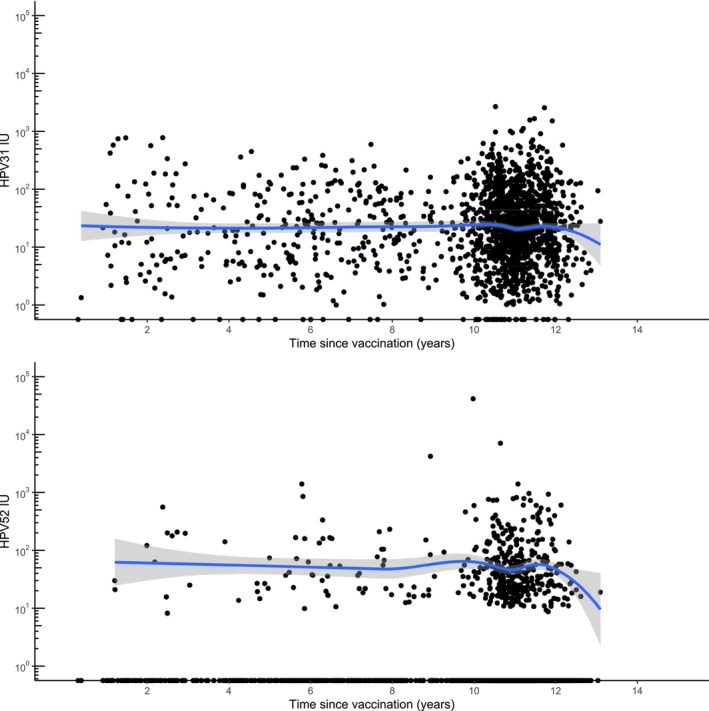
Human papillomavirus (HPV) type 33‐ and type 52‐specific L1 protein‐binding antibody levels (in international units) over time among HPV16/18‐vaccinated women participating in the Finnish community‐randomized vaccination and screening trials.^87^, ^89^

Interim results of this study were reported in the EUROGIN conference 2024: 1800 women were eligible for an interim analysis, with the majority having received HPV vaccination between ages 12 and 15 years. 97.4% were seropositive for binding antibodies to HPV31, while 56.3%, 33.9%, and 23.8% were seropositive for HPV45, 33, and 52, respectively. The HPV31/33/45/52 antibody levels were stable over 12 years postvaccination (Figure 2). There were 1, 2, and 0 breakthrough HPV31/33/45 infections but no such HPV16/18 infections. Among 23 women who acquired HPV52 in their subsequent sample, the geometric mean HPV52 antibody level was 0.91 IU (95% confidence intervals, 0.05–2.46 IU) compared to 1.46 IU (1.26–1.67 IU) among the women who remained HPV52‐negative. For HPV52, the antibody levels were lower in women with than without subsequent HPV52 infection but the confidence intervals overlapped. Cross‐reactive antibody threshold indicating the risk of HPV infections is now studied in the entire cohort.

## 7 CONCLUSIONS

Due to high HPV vaccine effectiveness against both vaccine‐type and related infections and disease, the WHO declared a Global strategy to accelerate the elimination of cervical cancer as a public health problem for which HPV vaccine coverage rates^93^ with associated herd protection,^92^ and correlates of protection will be key parameters. For Hepatitis B, a clear immune correlate of protection is defined as the antibody level after last vaccine injection and will protect, by immunologic memory mechanisms, even if the vaccinee has become seronegative.^3^ A similar strategy as used for HBV may be possible for HPV vaccine types. However, the high VE against vaccine HPV types necessitates to start by using protection against cross‐protected HPV types to define breakthrough cases, controls, and eventually the protective antibody levels. The required international standardization of HPV serology is accomplished, and longitudinal cohorts to enable addressing the issue exist. An important caveat that cross‐protection may not be mediated by the same antibody specificity as the immunodominant type‐specific immunity is being addressed by detailing of the vaccine‐induced antibody specificity. We are approaching a resolution of an important bottleneck in the work toward global HPV elimination.

## AUTHOR CONTRIBUTIONS

**Matti Lehtinen:** Conceptualization; writing – original draft; supervision. **Pierre van Damme:** Conceptualization; writing – review and editing; writing – original draft. **Simon Beddows:** Conceptualization; writing – review and editing; supervision; writing – original draft. **Ligia A. Pinto:** Conceptualization; writing – original draft; writing – review and editing. **Filipe Mariz:** Conceptualization; writing – original draft; writing – review and editing; investigation. **Penelope Gray:** Conceptualization; investigation; writing – original draft; writing – review and editing. **Joakim Dillner:** Conceptualization; writing – review and editing; supervision.

## CONFLICT OF INTEREST STATEMENT

ML has received grants for his HPV vaccination studies from Merck & Co. Inc. and GSK Biologicals.

## References

[1] Public assessment report. 2006. Accessed February 6, 2025. https://www.ema.europa.eu/PDFs/EPAR/gardasil/European

[2] Public assessment report. 2007. Accessed February 6, 2025. https://www.ema.europa.eu/PDFs/EPAR/cervarix/European

[3] Jack AD , Hall AJ , Maine N , Mendy M , Whittle HC . What level of hepatitis B antibody is protective? J Infect Dis. 1999;179:489‐492. 9878036 10.1086/314578

[4] Olakunde BO , Ifeorah IM , Adeyinka DA , et al. Immune response to hepatitis B vaccine among children under 5 years in Africa: a meta‐analysis. Trop Med Health. 2024;58:24. 10.1186/s41182-024-00594-4 PMC10983738 38561838

[5] Van Damme P , Leroux‐Roels G , Suryakiran P , et al. Persistence of antibodies 20 years after vaccination with a combined hepatitis a and B vaccine. Hum Vaccin Immunother. 2017;13:972‐980. 28281907 10.1080/21645515.2016.1274473 PMC5443376

[6] Gilca V , Sauvageau C , Boulianne N , et al. Immunogenicity of quadrivalent HPV and combined hepatitis a/B vaccine when co‐administered or administered one month apart to 9‐10 year‐old girls according to 0‐6 mo schedule. Hum Vaccin Immunother. 2014;10:2438‐2445. 25424952 10.4161/hv.29617 PMC4896778

[7] Zheng Z , Li G , Liao F , et al. Seroconversion of hepatitis B surface antigen among those with previously successful immune response in southern China. Hum Vac Immunother. 2021;17:845‐851. 10.1080/21645515.2020.1801076 PMC7996079 32961084

[8] Leuridan E , Van Damme P . Hepatitis B and the need for a booster dose. Clin Infect Dis. 2011;53:68‐75. 21653306 10.1093/cid/cir270

[9] Schiller J , Lowy D . Explanations for the high potency of HPV prophylactic vaccines. Vaccine. 2018;36:4768‐4773. 29325819 10.1016/j.vaccine.2017.12.079 PMC6035892

[10] Amanna IJ , Slifka MK . Mechanisms that determine plasma cell lifespan and the duration of humoral immunity. Immunol Rev. 2010;236:125‐138. 20636813 10.1111/j.1600-065X.2010.00912.x PMC7165522

[11] Ferguson M , Heath A , Johnes S , Pagliusi S , Dillner J , Collaborative Study Participants . Results of the first WHO international collaborative study on the standardization of the detection of antibodies to human papillomaviruses. Int J Cancer. 2006;118:1508‐1514. 16184553 10.1002/ijc.21515

[12] Ferguson M , Wilkinson DE , Zhou T . Who meeting on the standardization of the WHO Laboratory network in supporting vaccine introduction (Jan 24‐25, 2008). Vaccine. 2009;27:337‐347. 19007840 10.1016/j.vaccine.2008.10.062

[13] Pinto LA , Dillner J , Beddows S , Unger ER . Immunogenicity of HPV vaccines: serology assays and their use in HPV vaccine evaluation and development. Vaccine. 2018;36:4792‐4799. 29361344 10.1016/j.vaccine.2017.11.089 PMC6050153

[14] Tsanga SH , Basu P , Bender N , et al. Evaluation of serological assays to monitor antibody responses to single‐dose HPV vaccines. Vaccine. 2020;38:5997‐6007. 32713678 10.1016/j.vaccine.2020.07.017 PMC7429278

[15] Olsson S , Villa L , Costa R , et al. Induction of immune memory following administration of a quadrivalent HPV6/11/16/18 L1 virus‐like particle (VLP) vaccine. Vaccine. 2007;25:4931‐4939. 17499406 10.1016/j.vaccine.2007.03.049

[16] Arroyo S , Eklund C , Lagheden C , et al. Head‐to‐head comparison of bivalent and nonavalent human papillomavirus vaccine induced antibody responses. J Infect Dis. 2022;226:1195‐1199. 35535025 10.1093/infdis/jiac190 PMC9518834

[17] Gray P , Mariz F , Eklund C , et al. Absence of total and neutralizing human papillomavirus type 18 L1 antibodies every seventh quadrivalent vaccine recipient: a population‐based follow‐up study of the intervention arms of two phase 3 trials. NPJ Vaccines. 2024;9(1):146. doi:10.1038/s41541-024-00941-w 39138224 PMC11322158

[18] Joshi S , Anantharaman D , Muwange R , et al. Evaluation of immune response to a single dose of quadrivalent HPV vaccine at 10‐year post‐vaccination. Vaccine. 2023;41:236‐245. 36446654 10.1016/j.vaccine.2022.11.044 PMC9792650

[19] Mariz FC , Gray P , Bender E , et al. Sustainability of bi‐ and quadrivalent HPV vaccine‐induced neutralizing antibodies. Lancet Infect Dis. 2021;10:1458‐1468. 10.1016/S1473-3099(20)30873-2 34081923

[20] Beasley RP , Hwang LY , Lin CC , Chien CS . Hepatocellular carcinoma and hepatitis B virus: a prospective study of 22 707 men in Taiwan. Lancet. 1981;2:1129‐1133. 6118576 10.1016/s0140-6736(81)90585-7

[21] Blumberg BS , Alter HJ , Visnich S . A new antigen in leukemia sera. JAMA. 1965;191:541‐546. 14239025 10.1001/jama.1965.03080070025007

[22] Dane DS , Cameron CH , Briggs M . Virus‐like particles in serum of patients with Australia‐antigen‐associated hepatitis. Lancet. 1970;1:695‐698. 4190997 10.1016/s0140-6736(70)90926-8

[23] Edmunds WJ , Medley GF , Nokes DJ , et al. The influence of age on the development of the hepatitis B carrier state. Proc Biol Sci. 1993;253:197‐201. 8397416 10.1098/rspb.1993.0102

[24] European Centre for Disease Prevention and Control . Monitoring the Responses to Hepatitis B and C Epidemics in the EU/EEA Member States. European Centre for Disease Prevention and Control; 2019.

[25] World Health Organization . Hepatitis B vaccines. Wkly Epidemiol Rec. 2017;92:369.28685564

[26] Schweitzer A , Horn J , Mikolajczyk RF , et al. Estimations of worldwide prevalence of chronic hepatitis B virus infection: a systematic review of data published between 1965 and 2013. Lancet. 2015;386:1546‐1555. 26231459 10.1016/S0140-6736(15)61412-X

[27] Van Damme P , Ward J , Shouval D , et al. Hepatitis B vaccines. In: Plotkin SA , Orenstein WA , Offit PA , eds. Vaccines. 7th ed. Elsevier Sanders; 2017.

[28] Plotkin S . Recent updates on correlates of vaccine‐induced protection. Front Immunol. 2023;13:1081107. doi:10.3389/fimmu.2022.1081107 36776392 PMC9912984

[29] Jilg W , Schmidt M , Deinhardt F . Vaccination against hepatitis B: comparison of three different vaccination schedules. J Infect Dis. 1989;160:766‐769. 2530289 10.1093/infdis/160.5.766

[30] European Consensus Group on Hepatitis B Immunity . Are booster immunisations needed for lifelong hepatitis B immunity? Lancet. 2000;355:561‐565. 10683019

[31] Van Damme P , Vorsters A . Hepatitis B vaccines. In: Vesikari T , Van Damme P , eds. Paediatric Vaccines and Vaccination. 2nd ed. Springer; 2021.

[32] West D , Calandra GB . Vaccine induced immunologic memory for hepatitis B surface antigen: implications for policy on booster vaccination. Vaccine. 1996;14:1019‐1027. 8879096 10.1016/0264-410x(96)00062-x

[33] Plotkin S . Correlates of protection induced by vaccination. Clin Vaccine Immunol. 2010;17:1055‐1065. 20463105 10.1128/CVI.00131-10 PMC2897268

[34] Schonberger K , Riedel C , Ruckinger S , et al. Determinants of long‐term protection after hepatitis B vaccination in infancy: a meta‐analysis. Pediatr Infect Dis J. 2013;32:307‐313. 23249904 10.1097/INF.0b013e31827bd1b0

[35] Jilg W , Schmidt M , Deinhardt F . Four‐year experience with a recombinant hepatitis B vaccine. Infection. 1989;17:70‐76. 2714860 10.1007/BF01646879

[36] Centers for Disease Control and Prevention . Protection against viral hepatitis: recommendations of the advisory committee on immunization practices (ACIP). MMWR. 1990;39:1‐26. 2153904

[37] Pollard A , Bijker E . A guide to vaccinology: from basic principles to new developments. Nat Rev Immunol. 2021;21:83‐100. 33353987 10.1038/s41577-020-00479-7 PMC7754704

[38] Centers for Disease Control and Prevention . Prevention of hepatitis B virus infection in the United States: recommendations of the advisory committee on immunization practices. MMWR. 2018;67:1‐31. 10.15585/mmwr.rr6701a1 PMC5837403 29939980

[39] Cui F , Shen L , Wang H , et al. Prevention of chronic hepatitis B after 3 decades of escalating vaccination policy, China. Emerg Infect Dis. 2017;23:765‐772. 28418296 10.3201/eid2305.161477 PMC5403029

[40] Romano L , Paladini S , Van Damme P , et al. The worldwide impact of vaccination on the control and protection of viral hepatitis B. Dig Liver Dis. 2011;43(Suppl 1):S2‐S7. 21195368 10.1016/S1590-8658(10)60685-8

[41] Accessed July 9, 2024. https://www.who.int/health-topics/hepatitis/elimination-of-hepatitis-by-2030#tab=tab_1

[42] Consolidated guidelines on HIV . viral hepatitis and STI prevention, diagnosis, treatment and care for key populations. Accessed July 9, 2024. https://www.who.int/publications/i/item/9789240052390 2024. 36417550

[43] Accessed July 9, 2024. https://www.who.int/teams/global-hiv-hepatitis-and-stis-programmes/guidelines

[44] The European Vaccine Action Plan (2015–2020): Final Report. WHO Regional Office for Europe; 2024. Accessed July 9, 2024. https://www.who.int/europe/publications/i/item/WHO‐EURO‐2024‐9674‐49446‐73962

[45] Liu X , Chen J , Wanf Z , et al. Neutralization sites of human papillomavirus‐6 relate to virus attachment and entry phase in viral infection. Emerg Microbes Infect. 2019;8:1721‐1733. 31769733 10.1080/22221751.2019.1694396 PMC6883418

[46] Prabhu PR , Carter JJ , Galloway DA . B cell responses upon human papillomavirus (HPV) infection and vaccination. Vaccine. 2022;10:837. 10.3390/vaccines10060837 PMC9229470 35746445

[47] Yokoji K , Giguère K , Malagón T , et al. Association of naturally acquired type‐specific HPV antibodies and subsequent HPV re‐detection: systematic review and meta‐analysis. Infect Agent Cancer. 2023;18:70. 37941016 10.1186/s13027-023-00546-3 PMC10631102

[48] Pinto LA , Dillner J , Beddows S , Unger ER . Immunogenicity of HPV prophylactic vaccines: serology assays and their use in HPV vaccine evaluation and development. Vaccine. 2018;36:4792‐4799. 29361344 10.1016/j.vaccine.2017.11.089 PMC6050153

[49] Guan J , Bywaters SM , Brendle SA , et al. High‐resolution structure analysis of antibody V5 and U4 conformational epitopes on human papillomavirus 16. Viruses. 2017;9:374. 29211035 10.3390/v9120374 PMC5744149

[50] Godi A , Vaghadia S , Cocuzza C , Miller E , Beddows S . Contribution of surface‐exposed loops on the HPV16 capsid to antigenic domains recognized by vaccine or natural infection induced neutralizing antibodies. Microbiol Spectr. 2022;10:e0077922. 35475682 10.1128/spectrum.00779-22 PMC9241894

[51] Bishop B , Dasgupta J , Klein M , et al. Crystal structures of four types of human papillomavirus L1 capsid proteins: understanding the specificity of neutralizing monoclonal antibodies. J Biol Chem. 2007;282:31803‐31811. 17804402 10.1074/jbc.M706380200

[52] Burk RD , Harari A , Chen Z . Human papillomavirus genome variants. Virology. 2013;445:232‐243. 23998342 10.1016/j.virol.2013.07.018 PMC3979972

[53] Godi A , Kemp TJ , Pinto LA , Beddows S . Sensitivity of human papillomavirus (HPV) lineage and sublineage variant pseudoviruses to neutralization by Nonavalent vaccine antibodies. J Infect Dis. 2019;220:1940‐1945. 31412122 10.1093/infdis/jiz401 PMC6834066

[54] Kamuyu G , Coelho da Silva F , Tenet V , et al. Global evaluation of lineage‐specific human papillomavirus capsid antigenicity using antibodies elicited by natural infection. Nat Commun. 2024;15:1608. 38383518 10.1038/s41467-024-45807-w PMC10881982

[55] Shing J , Porras C , Pinheiro M , et al. Differential long‐term bivalent HPV vaccine cross protection by variants in the Costa Rica HPV vaccine trial. NPJ Vaccines. 2024;9:101. doi:10.1038/s41541-024-00896-y 38851816 PMC11162434

[56] Park I , Unger ER , Kemp TJ , Pinto LA . The second HPV serology meeting: progress and challenges in standardization of human papillomavirus serology assays. Vaccine. 2023;41:1177‐1181. 36642631 10.1016/j.vaccine.2023.01.008 PMC11216077

[57] Park I , Kemp TJ , Pinto LA . The HPV serology laboratory leads an initiative to standardize and harmonize human papillomavirus serology assays. PLoS Pathog. 2023;19:e1011403. 37384602 10.1371/journal.ppat.1011403 PMC10309594

[58] Kemp TJ , Panicker G , Eklund C , et al. WHO international standards for antibodies to HPV6, HPV11, HPV31, HPV33, HPV45, HPV52, and HPV58. NPJ Vaccines. 2024;9:165. 39256440 10.1038/s41541-024-00949-2 PMC11387505

[59] Miller C , Kemp TJ , Pinto LA . Development of a proficiency testing program for HPV serology assays used to evaluate antibody responses in vaccine trials. J Immunol Methods. 2023;523:113585. 37949349 10.1016/j.jim.2023.113585 PMC10841976

[60] WHO . WHO Manual for the Preparation of Reference Materials for Use as Secondary Standards in Antibody Testing. Report No.: 1043. WHO; 2022.

[61] Tsang SH , Basu P , Bender N , et al. Evaluation of serological assays to monitor antibody responses to single‐dose HPV vaccines. Vaccine. 2020;38:5997‐6006. 32713678 10.1016/j.vaccine.2020.07.017 PMC7429278

[62] Baisley K , Kemp TJ , Kreimer AR , et al. Comparing one dose of HPV vaccine in girls aged 9‐14 years in Tanzania (DoRIS) with one dose of HPV vaccine in historical cohorts: an immunobridging analysis of a randomised controlled trial. Lancet Glob Health. 2022;10:e1485‐e93. 36113532 10.1016/S2214-109X(22)00306-0 PMC9638025

[63] Baisley K , Kemp TJ , Mugo NR , et al. Comparing one dose of HPV vaccine in girls aged 9‐14 years in Tanzania (DoRIS) with one dose in young women aged 15‐20 years in Kenya (KEN SHE): an immunobridging analysis of randomised controlled trials. Lancet Glob Health. 2024;12:e491–9. 38365419 10.1016/S2214-109X(23)00586-7 PMC10882205

[64] Zeng Y , Moscicki AB , Woo H , et al. HPV16/18 antibody responses after a single dose of nonavalent HPV vaccine. Pediatrics. 2023;152:e2022060301. 37317810 10.1542/peds.2022-060301 PMC10312231

[65] Hempel HA , Kemp TJ , Roche N , et al. From HPV to COVID‐19 and beyond: leveraging the power of serology and standards. Lancet Microbe. 2023;4:e966–7. 37778361 10.1016/S2666-5247(23)00287-2

[66] Wong R , Huang H , Yu C , et al. Current status and future directions for the development of human papillomavirus vaccines. Front Immunol. 2024;15:1362770. 38983849 10.3389/fimmu.2024.1362770 PMC11231394

[67] Schiller JT , Castellsagué X , Garland SM . A review of clinical trials of human papillomavirus prophylactic vaccines. Vaccine. 2012;30(Suppl5):F123‐F138. doi:10.1016/j.vaccine.2012.04.108 23199956 PMC4636904

[68] Draper E , Bissett SL , Howell‐Jones S , et al. A randomized, observer‐blinded immunogenicity trial of Cervarix® and Gardasil® human papillomavirus vaccines in 12‐15 year old girls. PLoS One. 2013;8:e61825. doi:10.1371/journal.pone.0061825 23650505 PMC3641072

[69] Mariz FC , Bender N , Anantharama D , et al. Peak neutralizing and cross‐neutralizing antibody levels to human papillomavirus types 6/16/18/31/33/45/52/58 induced by bivalent and quadrivalent HPV vaccines. Npj Vaccines. 2020;5:14. doi:10.1038/s41541-020-0165-x 32128255 PMC7021830

[70] Artemchuk H , Eriksson T , Poljak M , et al. Long‐term antibody response to human papillomavirus vaccines: up to 12 years of follow‐up in the Finnish maternity cohort. J Infect Dis. 2019;219:582‐589. 30239832 10.1093/infdis/jiy545

[71] Paisley K , Kemp T , Kreimer AR , et al. Comparing one dose of HPV vaccine in girls aged 9‐14 years in Tanzania (DoRIS) with one dose of HPV vaccine in historical cohorts: an immunobridging analysis of a randomized controlled trial. Lancet Glob Health. 2022;10:1485‐1493. 10.1016/S2214-109X(22)00306-0 PMC9638025 36113532

[72] Watson‐Jones D , Changalucha J , Whitworth H , et al. Immunogenicity and safety of one‐dose human papillomavirus vaccine compared with two or three doses in Tanzanian girls (doris): an open‐label, randomised, non‐inferiority trial. Lancet Glob Health. 2022;10:e1473‐e1484. doi:10.1016/s2214-109x(22)00309-6 36113531 PMC9638030

[73] Tsang SH , Schiller JT , Porras C , et al. Hpv16 infection decreases vaccine‐induced anti‐body avidity: the CVT trial. NPJ Vaccines. 2022;7:40. doi:10.1038/s41541-022-00431-x 35351898 PMC8964739

[74] Roy V , Jung W , Linde C , et al. Differences in HPV‐specific antibody fc‐effector functions following Gardasil® and Cervarix® vaccination. NPJ Vaccines. 2023;8:39. doi:10.1038/s41541-023-00628-8 36922512 PMC10017795

[75] FUTURE II Study Group . Quadrivalent vaccine against human papillomavirus to prevent high‐grade cervical lesions. N Engl J Med. 2007;356:1915‐1927. 17494925 10.1056/NEJMoa061741

[76] Wheeler CM , Castellsagué X , Garland SM , et al. Cross‐protective efficacy of HPV‐16/18 AS04‐adjuvanted vaccine against cervical infection and precancer caused by non‐vaccine oncogenic HPV types: 4‐year end‐of‐study analysis of the randomised, double‐blind PATRICIA trial. Lancet Oncol. 2012;13:100‐110. 22075170 10.1016/S1470-2045(11)70287-X

[77] Malagón T , MacCosham A , Burchell AN , et al. Proportion of incident genital human papillomavirus detections not attributable to transmission and potentially attributable to latent infections: implications for cervical cancer screening. Clin Infect Dis. 2022;75:365‐371. 34849640 10.1093/cid/ciab985 PMC9427149

[78] Sankaranarayanan R , Prabhu PR , Pawlita M , et al. Immunogenicity and HPV infection after one, two, and three doses of quadrivalent HPV vaccine in girls in India: a multicentre prospective cohort study. Lancet Oncol. 2016;17:67‐77. 26652797 10.1016/S1470-2045(15)00414-3 PMC5357737

[79] Whitworth HS , Mounier‐Jack S , Choi EM , et al. Efficacy and immunogenicity of a single dose of human papillomavirus vaccine compared to multidose vaccination regimens or no vaccination: an updated systematic review of evidence from clinical trials. Vaccine X. 2024;19:100486. 38873638 10.1016/j.jvacx.2024.100486 PMC11169951

[80] Gargano JW , You M , Potter R , et al. An evaluation of dose‐related HPV vaccine effectiveness using central registries in Michigan. Cancer Epidemiol Biomarkers Prev. 2022;31:181‐193. 10.1158/1055-9965.EPI-21-0625 34663615

[81] Halloran ME , Longini IM , Struchiner CJ . Design and Analysis of Vaccine Studies (Statistics for Biology and Health). 10th ed. Springer; 2009.

[82] Longet S , Schiller JT , Bobst M , Jichlinski P , Nardelli‐Haefliger D . A murine genital‐challenge model is a sensitive measure of protective antibodies against human papillomavirus infection. J Virol. 2011;85:13253‐13259. 21976653 10.1128/JVI.06093-11 PMC3233130

[83] Quang C , Chung AW , Frazer IH , Toh ZQ , Licciardi PV . Single‐dose HPV vaccine immunity: is there a role for non‐neutralizing antibodies? Trends Immunol. 2022;43:815‐825. 35995705 10.1016/j.it.2022.07.011

[84] Day PM , Kines RC , Thompson CD , et al. In vivo mechanisms of vaccine‐induced protection against HPV infection. Cell Host Microbe. 2010;8:260‐270. 20833377 10.1016/j.chom.2010.08.003 PMC2939057

[85] Brown DR , Kjaer SK , Sigurdsson K , et al. The impact of quadrivalent human papillomavirus (HPV; types 6, 11, 16, and 18) L1 virus‐like particle vaccine on infection and disease due to oncogenic nonvaccine HPV types in generally HPV‐naive women aged 16‐26 years. J Infect Dis. 2009;199:926‐935. 19236279 10.1086/597307

[86] Hoes J , Pasmans H , Knol MJ , et al. Persisting antibody response 9 years after bivalent human papillomavirus (HPV) vaccination in a cohort of Dutch women: immune response and the relation to genital HPV infections. J Infect Dis. 2020;221:1884‐1894. 31917429 10.1093/infdis/jiaa007

[87] Lehtinen M , Apter D , Baussano I , et al. Characteristics of a cluster‐randomized phase IV human papillomavirus vaccination effectiveness trial. Vaccine. 2015;33:1284‐1290. 25593103 10.1016/j.vaccine.2014.12.019

[88] Lehtinen M , Söderlund‐Strand A , Vänskä S , et al. Impact of gender‐neutral or girls‐only vaccination against human papillomavirus‐results of a community‐randomized clinical trial (I). Int J Cancer. 2018;142:949‐958. 29055031 10.1002/ijc.31119

[89] Louvanto K , Eriksson T , Gray P , et al. Baseline findings and safety of infrequent vs. frequent screening of human papillomavirus vaccinated women. Int J Cancer. 2020;147:440‐447. 31749143 10.1002/ijc.32802

[90] Lehtinen M , Surcel HM , Natunen K , Pukkala E , Dillner J . Cancer registry follow‐up for 17 million person‐years of a nationwide maternity cohort. Cancer Med. 2017;12:3060‐3064. 10.1002/cam4.1222 PMC5727241 29071810

[91] Faust H , Knekt P , Forslund O , Dillner J . Validation of multiplexed human papillomavirus serology using pseudovirions bound to heparin‐coated beads. J Gen Virol. 2010;91:1840‐1848. 20181747 10.1099/vir.0.019349-0

[92] WHO . Global Strategy to Accelerate the Elimination of Cervical Cancer as a Public Health Problem. World Health Organization; 2020.

[93] Lehtinen M , Bruni L , Elfstöm M , et al. Scientific approaches towards improving cervical cancer elimination restrategies. Int J Cancer. 2024;154:1537‐1548. 38196123 10.1002/ijc.34839

